# Optical Genome Mapping: Integrating Structural Variations for Precise Homologous Recombination Deficiency Score Calculation

**DOI:** 10.3390/genes14091683

**Published:** 2023-08-25

**Authors:** Nikhil Shri Sahajpal, Ashis K. Mondal, Ashutosh Vashisht, Harmanpreet Singh, Andy Wing Chun Pang, Daniel Saul, Omar Nivin, Benjamin Hilton, Barbara R. DuPont, Vamsi Kota, Natasha M. Savage, Alex R. Hastie, Alka Chaubey, Ravindra Kolhe

**Affiliations:** 1 Greenwood Genetic Center, Greenwood, SC 29646, USA; nsahajpal@ggc.org (N.S.S.); bhilton@ggc.org (B.H.); dupont@ggc.org (B.R.D.); 2 Department of Pathology, Medical College of Georgia, Augusta University, Augusta, GA 30912, USA; amondal@augusta.edu (A.K.M.); avashisht@augusta.edu (A.V.); hsingh1@augusta.edu (H.S.); onivin@augusta.edu (O.N.); nsavage@augusta.edu (N.M.S.); 3 Bionano Genomics, San Diego, CA 92121, USA; apang@bionanogenomics.com (A.W.C.P.); dsaul@bionanogenomics.com (D.S.); ahastie@bionanogenomics.com (A.R.H.); achaubey@bionanogenomics.com (A.C.); 4 Department of Medicine, Medical College of Georgia, Augusta University, Augusta, GA 30912, USA; vkota@augusta.edu

**Keywords:** optical genome mapping, homologous recombination deficiency, HRD scores

## Abstract

Homologous recombination deficiency (HRD) is characterized by the inability of a cell to repair the double-stranded breaks using the homologous recombination repair (HRR) pathway. The deficiency of the HRR pathway results in defective DNA repair, leading to genomic instability and tumorigenesis. The presence of HRD has been found to make tumors sensitive to ICL-inducing platinum-based therapies and poly(adenosine diphosphate [ADP]–ribose) polymerase (PARP) inhibitors (PARPi). However, there are no standardized methods to measure and report HRD phenotypes. Herein, we compare optical genome mapping (OGM), chromosomal microarray (CMA), and a 523-gene NGS panel for HRD score calculations. This retrospective study included the analysis of 196 samples, of which 10 were gliomas, 176 were hematological malignancy samples, and 10 were controls. The 10 gliomas were evaluated with both CMA and OGM, and 30 hematological malignancy samples were evaluated with both the NGS panel and OGM. To verify the scores in a larger cohort, 135 cases were evaluated with the NGS panel and 71 cases with OGM. The HRD scores were calculated using a combination of three HRD signatures that included loss of heterozygosity (LOH), telomeric allelic imbalance (TAI), and large-scale transitions (LST). In the ten glioma cases analyzed with OGM and CMA using the same DNA (to remove any tumor percentage bias), the HRD scores (mean ± SEM) were 13.2 (±4.2) with OGM compared to 3.7 (±1.4) with CMA. In the 30 hematological malignancy cases analyzed with OGM and the 523-gene NGS panel, the HRD scores were 7.6 (±2.2) with OGM compared to 2.6 (±0.8) with the 523-gene NGS panel. OGM detected 70.8% and 66.8% of additional variants that are considered HRD signatures in gliomas and hematological malignancies, respectively. The higher sensitivity of OGM to capture HRD signature variants might enable a more accurate and precise correlation with response to PARPi and platinum-based drugs. This study reveals HRD signatures that are cryptic to current standard of care (SOC) methods used for assessing the HRD phenotype and presents OGM as an attractive alternative with higher resolution and sensitivity to accurately assess the HRD phenotype.

## 1. Introduction

Over the past decade, several poly (ADP-ribose) polymerase (PARP) inhibitors (PARPi) have been approved for use in ovarian, breast, prostate, and pancreatic cancers [1,2,3,4,5,6]. These agents inhibit PARP, which is a nuclear protein that acts as a DNA damage sensor and plays a role in DNA repair by recruiting DNA repair proteins [7,8]. The PARP inhibition results in the accumulation of double-strand breaks (DSB) that induces apoptosis, exploiting the mechanism of synthetic lethality, especially in cells with homologous recombination deficiency (HRD) [9]. Thus, it is essential to determine the presence of HRD in each tumor for appropriate therapy selection (candidates for PARPi) and prognostication.

For this reason, HRD is regarded as an emerging phenotype that is characterized by the inability of a cell to effectively repair DNA DSB using the homologous recombination repair (HRR) pathway [10]. The HRD is caused by various mechanisms that include: (a) Germline and somatic variants in BRCA1/2 genes, (b) Germline and somatic variants in homologous recombination repair (HRR) pathway genes, of which at least 24 HRR genes have been associated with HRD, (c) Promoter methylation of BRCA1/2 and other HRR pathway genes, and (d) Unknown causes [10,11]. Of these various mechanisms, BRCA1/2 gene variants have been consistently associated with the HRD phenotype, with clinical implications in ovarian, breast, prostate, and pancreatic cancers. However, the other HRR pathway gene associations were found to be less consistent and have unclear clinical implications [12]. Nevertheless, the impaired HRR pathway, irrespective of causative mechanism, results in certain genomic aberrations related to DSB repair and a characteristic genomic pattern or signature that can be quantitated and associated with the HRD phenotype [10,13,14,15].

Three independent signatures (HRD scars) of genomic instability have been used to predict the HRD phenotype. These three signatures include loss of heterozygosity (LOH) of regions that are >15 MB and <whole chromosome [16], telomeric allelic imbalance (TAI), i.e., regions with an allelic imbalance that extend to the sub-telomere but do not cross the centromere [17], and large-scale state transitions (LST) that are chromosome breaks (translocations, inversions, insertions, duplications, or deletions) of >10 MB and <whole chromosome [18]. Recently, a summation of all three signatures has been reported as the most sensitive method to determine the HRD phenotype [10,13,14]. The HRD phenotype determined using HRD score calculations has shown promise in breast and ovarian cancers, and is demonstrating high sensitivity to identify HR-deficient tumors in other tumor types, such as lung cancers [19].

However, regardless of the test(s) used to estimate HRD, for example, MGMT promoter hyper-methylation and/or IDH status (gliomas, myeloid neoplasms) [20,21,22], specific rearrangement (gene fusions), or accessing the HRD score [10,13,14,19], these tests lack negative predictive value and inadequately address the complex nature of the HRD phenotype. As a result, these tests consistently fail to explain a subgroup of selected patients who do not respond to PARPi. For HRD score calculations, one plausible explanation would be the limited sensitivity of current tools to adequately capture the HRD scars that would lead to lower specificity in identifying patients that could derive benefit from PARPi therapy. In this study, we highlight the application of optical genome mapping (OGM) to calculate HRD scores, using two cancer types, gliomas (for benchmarking with chromosomal microarray) and hematological malignancies, leveraging the three already established HRD scar classes. We highlight the structural variants that are intractable to current technologies that OGM can leverage to calculate HRD scores and propose that missing these variants using traditional methods might explain the lack of good correlation of HRD scoring with PARPi sensitivity. This study aimed to highlight the magnitude of genomic instability resulting from HRD that is beyond the detection capabilities of current technologies used for HRD scoring. As over 150 clinical trials are currently underway to evaluate HRD in various tumor types, it is important to ask the question of whether we are using the right tool for HRD calculation.

## 2. Materials and Methods

### 2.1. Sample Selection

This retrospective study included the analysis of 196 samples, of which 10 were gliomas, 176 were hematological tumors, and 10 were controls. The 10 gliomas were evaluated with chromosomal microarray (CMA) (OncoScan or CytoScan, ThermoFisher Scientific, Waltham, MA, USA) and OGM (Bionano Genomics, San Diego, CA, USA). Of the 176 hematological tumor samples, 135 were evaluated with a 523-gene next-generation sequencing (NGS) panel (Illumina, San Diego, CA, USA), 71 were evaluated with OGM, while 30 samples were common (evaluated with both technologies). The 176 hematological tumor samples comprised of: Acute myeloid leukemia = 79, Myelodysplastic neoplasms = 47, Acute undifferentiated leukemia = 2, B-cell acute lymphoblastic leukemia = 3, Chronic lymphocytic leukemia = 16, Chronic myeloid leukemia = 4, Chronic myelomonocytic leukemia = 2, Cytopenias = 2, Essential thrombocythemia = 2, Eosinophilia = 1, Erythrocytosis = 1, Lymphoid = 3, Leukopenia = 1, Lymphoma = 1, Lymphoplasmacytic Lymphoma = 1, Myeloid Neoplasm = 7, Myeloproliferative neoplasms = 12, Neutropenia = 1, Plasma Cell Myeloma = 10, Thrombocytopenia = 3, Polycythemia = 1, T-cell acute lymphoblastic leukemia = 1, Thrombocytosis = 4. The study was performed under IRB A- BIOMEDICAL I (IRB REGISTRATION #00000150), Augusta University. HAC IRB # 611298. Based on the IRB approval, the need for consent was waived; all PHI was removed, and all data were anonymized before accessing the clinical validation study.

### 2.2. Optical Genome Mapping

Ultra-high molecular weight (UHMW) DNA was isolated, labeled, and processed for analysis on the Bionano Genomics Saphyr^®^ platform following the manufacturer’s protocols (Bionano Genomics, San Diego, CA, USA). Briefly, 30 mg of tumor tissue or 1.5 M cells from bone marrow aspirate (BMA) were digested with Proteinase K (PK) and lysed using Lysis and Binding Buffer (LBB). DNA was precipitated on a nanobind magnetic disk using isopropanol and washed using buffers (buffers A and B). The UHMW-bound DNA was suspended in an elution buffer and quantified using Qubit broad range (BR) dsDNA assay kits (ThermoFisher Scientific, San Francisco, CA, USA).

DNA labeling was performed following manufacturer’s protocols (Bionano Genomics, San Diego, CA, USA) in which 750 ng of purified UHMW DNA was labeled at a specific 6-base sequence motif with DL-green fluorophores using Direct Labeling Enzyme 1 (DLE-1) reactions. Following the labeling reaction, the DLE enzyme was digested using PK and the DL-green was removed in two steps using an adsorption membrane in a micro-titer plate. Finally, the DNA backbone was stained blue using DNA stain and quantified using Qubit high sensitivity (HS) dsDNA assay kits. Labeled DNA was loaded onto flow cells of Saphyr chips for optical imaging. The fluorescently labeled DNA molecules were imaged on the Saphyr platform after the labeled DNA molecules were electrophoretically linearized in the nanochannel arrays. Analytical QC targets were set to achieve >300× effective coverage of the genome, >70% mapping rate, 13–17 label density (labels per 100 kbp), and >230 kbp N50 (of molecules >150 kbp).

### 2.3. Structural Variation Analysis Using OGM

Genome analysis was performed using the rare variant pipeline included in the Bionano Access (v.1.6 or v.1,7)/Bionano Solve (v.3.6 or v.3.7) software for all the samples. Briefly, single molecules were directly aligned against the reference genome (GRCh38) to detect SVs (insertion, duplications, deletions, inversions, and translocations) based on the differences in the alignment of labels between the sample and the reference assembly. Additionally, a coverage-based algorithm enabled the detection of large CNVs and aneuploidies.

For data analysis, the variants were filtered using the following criteria: (1) The manufacturer’s recommended confidence scores were applied: insertion: 0, deletion: 0, inversion: 0.01, duplication: −1, translocation: 0, and copy number: 0.99 (low stringency, filter set to 0); (2) The GRCh38 SV mask filter that hides any SVs in difficult-to-map regions was turned off for analysis; (3) To narrow the number of variants to be analyzed, we filtered out polymorphic variants, i.e., filtered out variants that appeared in >0% of an internal OGM control database (n > 300).

### 2.4. HRD Scoring Using Automated SV Scripts (OGM Data)

The OGM HRD-calculation script accepts an annotated SV file (.smap), a CNV file, and an aneuploidy file generated by the Access 1.7 or Solve 3.7 rare variant pipeline. It first extracts high-confidence variants above the OGM’s recommended variant confidence scores, rare SVs not seen in a user-defined percentage (by default zero percent) in the OGM control SV database, and SVs with molecule coverage support (by default at least five molecules spanning SV breakpoints). To handle fragmented CNV calls, the script also conjoins or stitches adjacent (by default within 500 kbp) variants, and when defining centromeric and telomeric regions, it includes the surrounding CNV masked regions, where the CNV-calling algorithm has reduced sensitivities. The script calculates HRD scores per chromosome based on the scheme outlined above. Finally, the script highlights chromosomes that could be affected by chromothripsis, which is defined as having more than a default of 15 intrachromosomal fusion events. Users can subsequently consider whether to include the HRD values from these highly rearranged chromosomes. The script is available at https://github.com/bionanogenomics/HRD (accessed on 24 August 2023).

### 2.5. Chromosomal Microarray

The DNA isolated for optical genome mapping was used for chromosomal microarray analysis following the manufacturer’s protocol (OncoScan^®^ FFPE assay kit, ThermoFisher Scientific, Waltham, MA, USA). The platform consists of 220,000 markers throughout the entire genome. The test compares the samples to control samples from the HAPMap set of 270 individuals. The raw data were analyzed using the NxClinical software v6.1 software and were matched to in silico reference sets.

### 2.6. 523-Gene Next-Generation Sequencing (NGS) Panel

DNA was isolated from BMA using the QIAamp DNA Blood Mini kit (QIAGEN, Hilden, Germany) as per the manufacturer’s protocol. Double-stranded DNA was measured using a Qubit dsDNA broad-range assay kit (#Q32850, Invitrogen, Waltham, MA, USA) and 120 ng gDNA was used for library preparation. The libraries were prepared using the hybrid capture-based TSO 500 library preparation kit (# 20028214, TruSight Oncology 500 DNA Kit, Illumina, San Diego, CA, USA) following the manufacturer’s instructions. In brief, the DNA was fragmented using an ultrasonicator (Covaris, Woburn, MA, USA) with a target peak of ~130 bp. After end repair, A-tailing, and adapter ligation, the adapter-ligated fragments were amplified using index PCR (UP-index) specific primers. Further, the libraries were enriched through a hybrid capture-based method using specific probes. This was followed by PCR-based enrichment, cleanup, and quantification of double-stranded DNA using high-sensitivity Qubit (#Q32854 Invitrogen, Waltham, MA, USA) measurement. The libraries were subjected to bead-based normalization and were sequenced using V2 sequencing reagent kits on a NextSeq550 platform (Illumina, San Diego, CA, USA) as per manufacturer recommendations.

### 2.7. NGS Variant Calling and Data Analysis

The raw sequence reads FASTQ files were converted to BAM and VCF files using Qiagen clinical insight interpret (QCI-I) clinical decision support software (Qiagen, Germantown, MD, USA). The BAM was utilized to call CNVs in NxClinical v.6.1 using the baseline sequencing reads throughout the genome.

### 2.8. Homologous Recombination Deficiency (HRD) Scoring

The HRD scores were calculated using the below definitions in NxClinical software v.1.7 for CMA and the 523-gene NGS panel, using a decision tree to automatically calculate the HRD scores. The HRD score for OGM was calculated using Bionano access software v.1.6 using the same definitions.

The signatures were defined as:

1. HRD-LOH: number of regions’ with losses and absence of heterozygosity (AOH) > 15 MB (not whole chromosome);
2. TAI: number of regions’ with gains, losses, and AOH > 3 MB from telomere (not involving the centromere);
3. TAI-LOH: Meets the definition of both HRD-LOH and TAI;
4. LST: number of breakpoints between gains, losses, and AOH segments > 10 MB, exclusive of intervals < 3 MB (not accounting for centromeric breaks).

The scoring was defined as:

1. TAI-0 = terminal gains, losses, and AOH > 3 MB < 10 MB (score = 1);
2. TAI-1 = terminal gains, losses, and AOH > 10 MB (score = 2);
3. TAI-LOH-1 = losses and LOH > 15 MB involving telomeres (score = 3);
4. LOH-1 = interstitial losses and AOH > 15 MB with anticipated 1 chromosome breakpoint (bp) (score = 2);
5. LOH-2 = interstitial losses and AOH > 15 MB with anticipated 2 chromosome bp (score = 3);
6. LOH-0 = interstitial losses and AOH > 15 MB with anticipated 0 chromosome bp (score = 1);
7. LST-1 = interstitial gains, losses, and AOH > 10 MB with anticipated 1 chromosome bp (score = 1);
8. LST-2 = interstitial gains, losses, translocations, and intra-chromosomal rearrangements and AOH > 10 MB with anticipated 2 chromosome bp (score = 2);
9. LST-3 = insertions > 10 MB with anticipated 3 chromosome bp (score = 3).

## 3. Results

### 3.1. OGM Data QC Metrics

All samples achieved the minimum QC metrics of >300× effective coverage of the genome, >70% mapping rate, 13–17 label density (labels per 100 kbp), and >230 kbp N50 (of molecules >150 kbp).

### 3.2. Validation of OGM Automated Scoring

**Figure 1:**
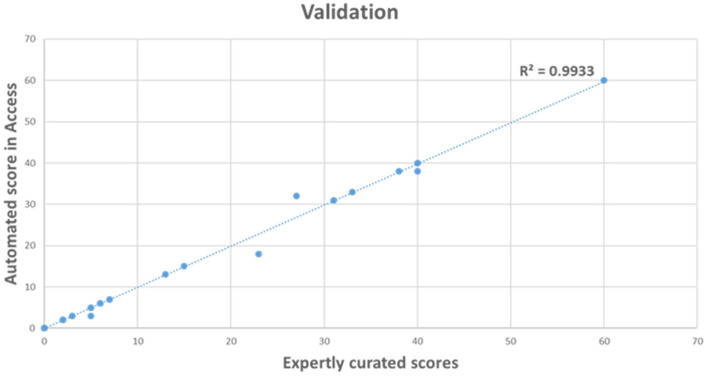
Validation of automated scoring with Access compared to expertly curated scores for HRD calculation using Optical Genome Mapping.

A total of 40 samples were used to validate the OGM automated tool for HRD calculation. The 40 samples included 10 gliomas, 20 hematological malignancies, and 10 control samples. The score from the automated tool was compared against expertly curated HRD scores. The results were 99.3% concordant between automated and expertly curated scores (Figure 1).

### 3.3. Comparison of HRD Scores between OGM and CMA in Gliomas

**Figure 2:**
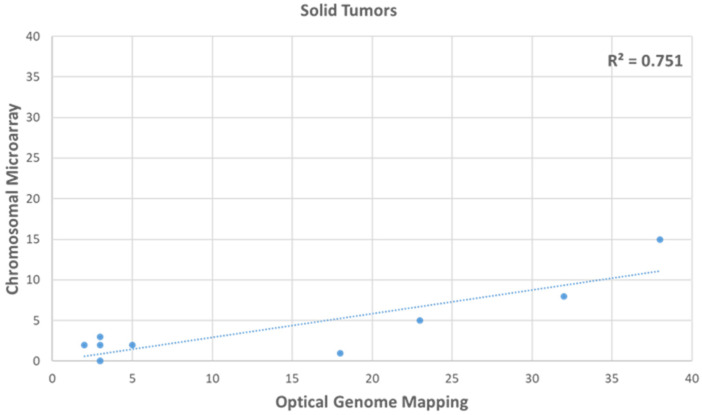
Comparison of HRD score calculated with CMA compared to OGM in 10 glioma samples.

In the ten glioma cases analyzed with OGM and CMA using the same DNA (to remove any tumor percentage bias), the HRD scores were 13.2 (±4.2) with OGM compared to 3.7 (±1.4) with CMA. OGM was 86.5% concordant with CMA in detecting the HRD scars, missing two absences of heterozygosity regions in one case. However, OGM detected 70.8% of additional structural variants that resulted from HRD, which included translocations, inversions, and fusions (Figure 2).

**Figure 3:**
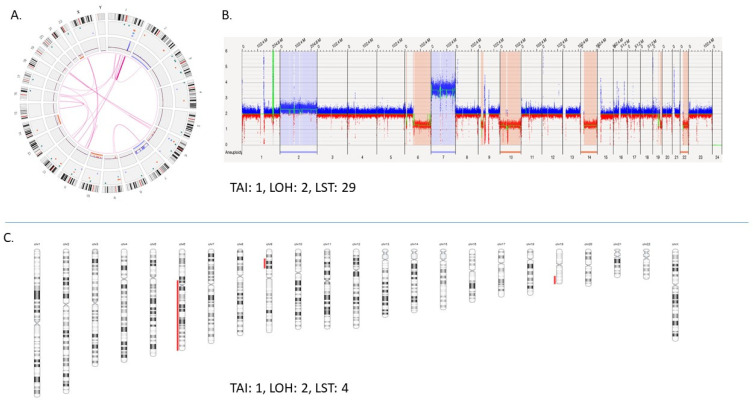
Shows the visualization and comparison of HRD scores for a case of glioblastoma with optical genome mapping and chromosomal microarray. (**A**) Optical genome mapping: shows the circos plot with an overview of the structural variations, copy number variations, aneuploidy, translocations, and fusions in the sample. (**B**) Optical genome mapping: whole genome CNV view showing region with copy number gain (blue columns), and copy number loss (red columns). (**C**) Chromosomal microarray: HRD scars visualization in NxClinical using the decision tree with HRD scar and scoring filtration.

**Figure 4:**
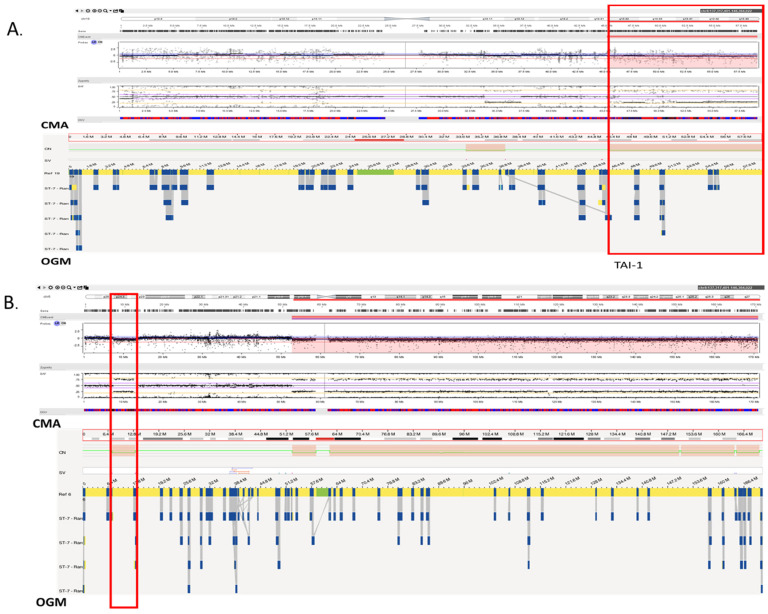
Shows the concordant call between optical genome mapping and chromosomal microarray. (**A**) Shows a >10 Mb <15 Mb copy number loss, resulting in TAI-1 (HRD score = 2). (**B**) Shows a <10 Mb copy number loss, which did not add to HRD score (HRD score = 0) (highlighted in red box).

**Figure 5:**
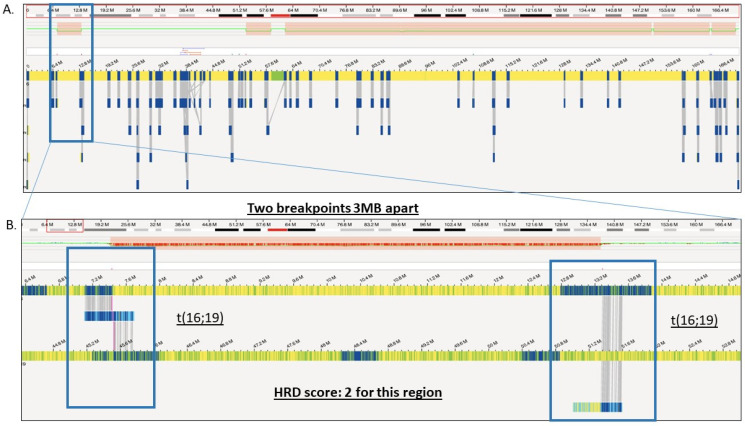
Shows the zoomed-in view of Figure 4B (<10 Mb) copy number loss with optical genome mapping. (**A**) Shows the 5.3 Mb deletion. (**B**) Zoomed-in view of the 5.3 Mb deletion showing translocations t(16;19) at both breakpoints (highlighted in blue boxes). This segment resulted in a HRD score of 2 with OGM, and a HRD score of 0 with CMA.

The difference in HRD scores is illustrated with an example where, in a case of glioma, the HRD score with OGM was 32 comprising of 29 LST, 2 LOH, and 1 TAI compared to a score of 7 with CMA comprising of 4 LST, 2 LOH, and 1 TAI (Figure 3). The concordance between the two platforms can be visualized in Figure 4A,B where both platforms detected the LOH-1 (>15 MB deletion including centromere with 1 bp) on chromosome 6 (Figure 4A). However, OGM detected several additional HRD scars, where a small deletion (5.3 Mb) was detected with both platforms (Figure 4D), but only contributed to HRD scoring with OGM as OGM detected that the deletion resulted from two translocations yielding a score of 2 for that region of the genome (Figure 5A,B).

### 3.4. Comparison of HRD Scores between OGM and 523-Gene NGS Panel in Hematological Malignancy Cases

**Figure 6:**
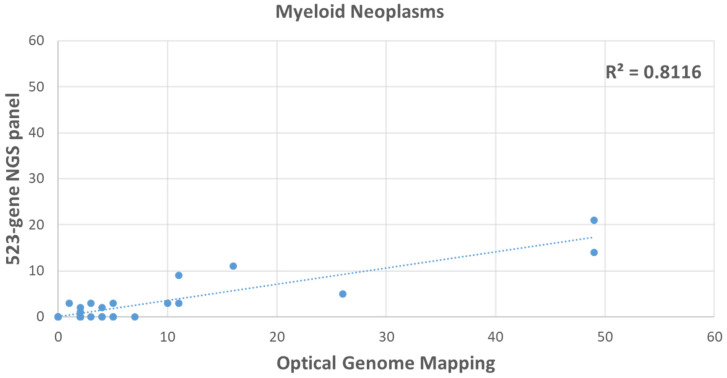
Comparison of HRD score calculated with the 523-gene NGS panel compared to OGM in 30 hematological malignancy samples.

**Figure 7:**
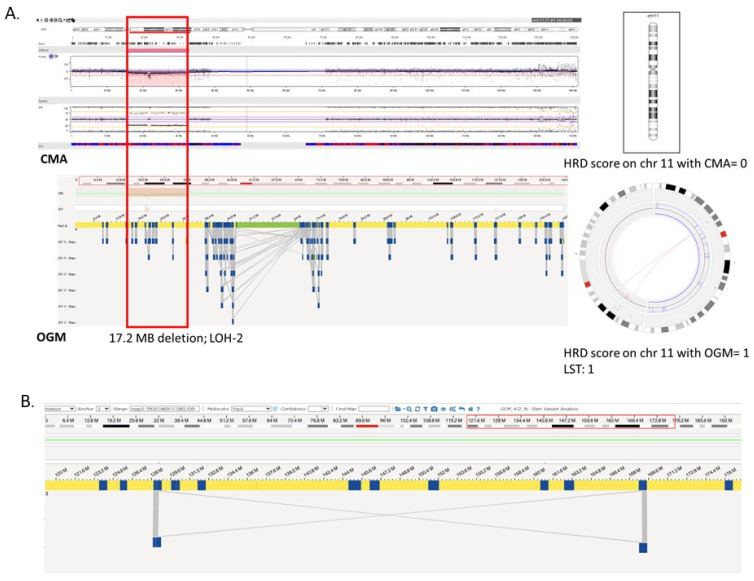
Additional structural variations detected with OGM that result from HRD. (**A**) Shows the comparison of OGM with CMA for a HRD scar, LOH-2 (>15 Mb deletion), that contributed a score of 3 with CMA and 4 with OGM as the deletion was part of a translocation t(9;11) (highlighted in red box). (**B**) Shows a large (>10 Mb) inversion with OGM that contributes a score of 2 towards HRD calculation.

In the 30 hematological malignancy cases analyzed with OGM and the 523-gene NGS panel, the HRD scores were 7.6 (±2.2) with OGM compared to 2.6 (±0.8) with the 523-gene NGS panel. OGM detected 66.8% additional structural variants that resulted from HRD, which included translocations, inversions, and fusions (Figure 6 and Figure 7). Importantly, similar trends were found when the scores were calculated in a higher number of samples, 7.8 (+1.6) in 71 samples with OGM and 1.3 (±0.27) in 135 samples with the 523-gene NGS panel. These scores reflect that the samples used for comparison with both technologies were representative and affirm the robustness of the findings.

**Figure 8:**
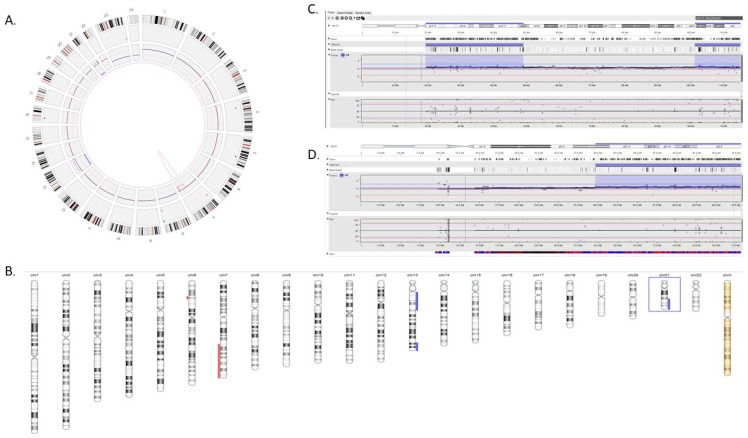
Shows the comparison between optical genome mapping and the 523-gene NGS panel in a case of acute myeloid leukemia. (**A**) Circos plot with OGM, showing an interstitial deletion at 7q, and trisomies 13 and 21. (**B**) Shows the HRD scarring in NxClinical using 523-gene panel data. (**C**) Zoomed-in view of chromosome 13 showing two interstitial regions with gains (TAI-1 and LST-1) and limited coverage to assist the analyst to determine true/false positive calls. (**D**) Zoomed-in view of chromosome 21 showing a TAI-1. Both segmental calls at chromosomes 13 and 21 were determined to be false calls, as these were trisomies as observed with OGM and confirmed with karyotyping.

Apart from the additional HRD scars detected with OGM, we want to highlight the potential false positive results with the 523-gene panel in HRD calculation. In the case of AML, OGM detected a LOH-2 (>15 Mb deletion with 2 bp), and trisomies 13 and 21. The results were concordant with karyotyping. However, the 523-gene NGS panel detected two distinct copy number gains on chromosome 13 (TAI with 1 bp and 1 LST) and a copy number gain of chromosome 21 (TAI with 1 bp), yielding a score of 5, whereas these segmental calls were due to the mosaicism and limited coverage on these chromosomes. These attributes make it difficult for the analyst to decide if this is aneuploidy or segmental CNVs (Figure 8A–D). 

## 4. Discussion

Genomic instability is a hallmark feature of tumorigenesis and cancer [23]. The genetic aberrations have a characteristic signature that reflects defects in specific DNA repair mechanisms. Recently, three independent signatures (HRD scars) of genomic instability that include, LOH, LST, and TAI have been used to predict a HRD phenotype (impaired HRR pathway), as demonstrated in breast and ovarian cancers [10,13,14]. The high success rate of PARPi in breast and ovarian cancers might be attributed to a subset of these cases enriched with *BRCA1/2* pathogenic variants and a sensitive threshold to distinguish HR deficient and HR proficient tumors [13,14]. The results in these tumors have been encouraging and have led to the initiation of several clinical trials to evaluate the sensitivity of PARPi in different tumor types. Given that the tumors with the HRD phenotype appear to be more sensitive to PARPi, it is important to accurately detect these tumors with high sensitivity and specificity. However, several tumor types do not show *BRCA1/2* mutations, and the genomic instability in those tumors is marked by translocations and fusions (gliomas and hematological malignancies) compared to copy number changes. In such tumors, the tools that are currently being used might not be ideal to calculate HRD scores to determine the HRD phenotype. In this pilot study, the sensitivity of different platforms was evaluated to determine the most sensitive tool to detect genetic aberrations that result from HRD.

For HRD score benchmarking, the HRD scores were compared between CMA and OGM in gliomas. The genomic instability in the glioma cases in this study was typically marked by translocations and fusions. As a result, OGM detected 77.2% additional structural variants compared to CMA resulting from HRD. The gliomas have shown significant sensitivity to PARPi in preclinical studies [24] but the same has not translated into clinical studies, though a subset of gliomas responds to PARPi. PARPi has been classically used to sensitize the tumors to temozolomide [25]. Recently, the PDX preclinical trial has identified MGMT promoter methylation as a marker for PARPi sensitivity, specifically veliparib-mediated sensitization of the tumors [26]. Additionally, the *IDH1/2* mutated gliomas have also shown sensitivity to PARPi and as a result, several of the current clinical trials are aimed at evaluating PARPi sensitization of gliomas to temozolomide or combinatorial drug treatments (including PARPi) using either MGMT promoter methylation or *IDH1/2* mutation status to stratify patients that can benefit from these combination therapies [25]. However, the selection of MGMT promoter methylation or *IDH1/2* mutation tumors would identify only a subset of tumors as tumors with HRD due to other mechanisms that would not be detected with this approach. In a study by Krutz et al., the patients with high-grade gliomas were stratified as High-HRD (>5% LOH or >3% LOH) and Low-HRD (<5% LOH or <3% LOH), and used the Kaplan–Meier method to assess OS and PFS, with a weak association between LOH score and *IDH* status (*p* = 0.09) [27]. These studies highlight the need for further investigations to determine the most sensitive methods to assess the HRD phenotype. Given that, the HRD scores in breast and ovarian cancers showed high sensitivity and specificity for response to PARPi, and the lack of association in gliomas warrants the use of OGM to calculate HRD scores to determine the HRD phenotype.

Importantly, there has been a significant interest in evaluating PARPi in hematological malignancies. Four clinical trials have completed phase I, of which two trials used single-agent PARPi while the other two were conducted to evaluate combination therapies. The single-agent PARPi was well-tolerated but resulted in modest clinical efficacy, and although the combination therapy showed improved results, these clinical trials were conducted on unselected patients. An HR-deficient tumor selection process might have shown improved sensitivity to PARPi. Currently, at least four clinical trials are underway which are based on selecting patients with specific variants. Of these, two clinical trials aimed at evaluating single-agent PARPi, talazoparib in cohesin-mutant AML (NCT03974217), and olaparib in *IDH1/2* mutant AML are in phase I and II, respectively [22]. While other biomarkers such as *BCR::ABL*, *RUNX1::RUNX1T1*, *PML::RARA*, and *MGMT* promoter methylation are being explored, there is a need to consider and evaluate genomic instability (HRD scars) as a biomarker for hematological malignancies, given it has shown promising results in identifying HR-deficient tumors in ovarian and breast cancers.

Notably, the hematological malignancy samples are enriched with translocations and fusions, relative to CNVs, and might be one reason that HRD scars might have remained of limited utility in hematological malignancies. OGM detected several translocations and fusions (Figure 5), detecting 65% of additional structural variations resulting from HRD. Given the clinical utility of OGM as a SOC method for cytogenetic analysis of hematological malignancies, and the higher sensitivity to detect HRD scars, it would be clinically relevant and very interesting to evaluate the HRD phenotype with OGM, especially as these tumors seem to be driven by chromosomal rearrangements. Overall, this pilot study aims to initiate this debate regarding the right tools that should be used for HRD calculation. Several groups have demonstrated the higher sensitivity of OGM in different applications [28,29,30,31], and this study highlights its utility as a highly sensitive method that can capture the HRD scars compared to the tools that are currently being used for HRD calculations. OGM has higher resolution and sensitivity to detect several classes of structural variants as compared to CMA and copy number calling from 523-gene NGS panels. CMA and 523-gene NGS panels cannot detect balanced events, including translocations, insertions, and inversions. Additionally, in unbalanced events such as unbalanced translocations, CMA can only detect copy number changes and cannot detect if those resulted from translocations. As demonstrated in the glioblastoma case, where a 5.3 Mb deletion was excluded from HRD scoring from CMA, the same resulted in a score of 2 with OGM as the deletion resulted from two translocations. Thus, OGM presents several advantages over currently used methods used for HRD calculations, as it can detect copy number changes and balanced and unbalanced events in the same assay (translocations, insertions, and inversions). It is also noteworthy that another approach to assess HRD status using OGM has recently been shown by Vanhuele et al., where insertion and tandem duplications captured with OGM analysis can discriminate HRD breast tumors from HR proficient tumors [32].

In conclusion, the current literature highlights the high variability in the platforms and the definition of HRD scars that are being used to calculate HRD. Further, different studies have calculated different HRD score thresholds to characterize tumors as HR deficient or HR proficient even in the same tumor types. For example, Telli et al. have reported a cutoff of >42 [33], while Min et al. have reported a cutoff of 57 in breast tumor [34]. The lack of uniformity regarding platforms, HRD scars, HRD thresholds used, and test type used (FDA-approved tests and lab-developed tests) highlights the lack of consensus regarding the HRD phenotype. In this phase, when several clinical trials are underway or are being initiated, it is necessary to use the most sensitive tools that can capture the phenotype accurately. This study highlights the HRD scars that are missed by current technologies used for accessing the HRD phenotype and presents OGM as an alternative tool with high resolution and sensitivity to most accurately assess the HRD phenotype.

## Data Availability

All relevant data in provided in the manuscript. The script for HRD score calculation is available at https://github.com/bionanogenomics/HRD (accessed on 24 August 2023).
